# Overexpression and characterization of medium-chain-length polyhydroxyalkanoate granule bound polymerases from *Pseudomonas putida *GPo1

**DOI:** 10.1186/1475-2859-8-60

**Published:** 2009-11-19

**Authors:** Qun Ren, Guy de Roo, Bernard Witholt, Manfred Zinn, Linda Thöny-Meyer

**Affiliations:** 1 Laboratory for Biomaterials, Swiss Federal Laboratories for Materials Testing and Research (Empa), CH-9014 St. Gallen, Switzerland; 2 Synthon BV, P.O. BOX 7071, 6503 GN Nijmegen, the Netherlands; 3 Institute of Molecular Systems Biology, Swiss Federal Institute of Technology, CH-8093 Zurich, Switzerland

## Abstract

**Background:**

Polyhydroxyalkanoates (PHA) are synthesized by many bacteria in the cytoplasm as storage compounds for energy and carbon. The key enzymes for PHA biosynthesis are PHA polymerases, which catalyze the covalent linkage of 3-hydroxyacyl coenzymeA thioesters by transesterification with concomitant release of CoA. *Pseudomonas putida *GPo1 and many other *Pseudomonas *species contain two different class II polymerases, encoded by *phaC1 *and *phaC2*. Although numerous studies have been carried out on PHA polymerases and they are well characterized at the molecular level, the biochemical properties of the class II polymerases have not been studied in detail. Previously we and other groups purified the polymerases, however, the activities of the purified enzymes were several magnitude lower than the granule-bound enzymes. It is problematic to study the intrinsic properties of these enzymes with such low activities, although they are pure.

**Results:**

PHA polymerase 1 (PhaC1) and PHA polymerase 2 (PhaC2) from *P. putida *GPo1 were overexpressed in the PHA-negative host *P. putida *GPp104 and purified from isolated PHA granules. Only minor activity (two to three orders of magnitude lower than that of the granule bound proteins) could be recovered when the enzymes were purified to homogeneity. Therefore, kinetic properties and substrate ranges were determined for the granule bound polymerases. The polymerases differed significantly with respect to their association with PHA granules, enzyme kinetics and substrate specificity. PhaC2 appeared to bind PHA granules more tightly than PhaC1. When *R*-3-hydroxyoctanoic acid was used as substrate, the granule-bound PhaC1 exhibited a *Km *of 125 (± 35) μM and a *V*max of 40.8 (± 6.2) U/mg PhaC1, while a *Km *of 37 (± 10) μM and a *V*max of 2.7 (± 0.7) U/mg PhaC2 could be derived for the granule-bound PhaC2. Granule-bound PhaC1 showed a strong preference for medium chain length (mcl-) 3-hydroxyacly-CoAs, with highest affinity towards 3-hydroxydecanoyl-CoA (40 U/mg PhaC1). Granule-bound PhaC2 demonstrated a far broader specificity ranging from short chain length up to long chain length substrates. Activity increased with increasing chain length with a maximum activity for 3-hydroxyacyl-CoAs containing 12 or more C-atoms.

**Conclusion:**

The kinetic properties and substrate ranges were determined for both granule bound polymerases. Evidence was provided for the first time that two PHA polymerases exhibited significant differences in granule release and in vitro activity profiles, suggesting that there are substantial functional differences between granule bound PhaC1 and PhaC2.

## Background

Many bacteria are able to accumulate polyhydroxyalkanoates (PHA) in the cytoplasm as storage compounds for energy and carbon [1]. The key enzymes for PHA biosynthesis are PHA polymerases. These enzymes catalyze the covalent linkage of 3-hydroxyacyl coenzyme A (CoA) thioesters by transesterification with concomitant release of CoA. PHA polymerases comprise a new family of enzymes with unique features, particularly when considering their functional role in the biogenesis of the water-insoluble subcellular structures of PHA granules. About 50 different PHA polymerases have been cloned and found to have an overall amino acid identity of 21-28% with only eight residues which are strictly conserved [2,3]. With respect to size, structure and substrate specificity, four different types of PHA polymerases can be distinguished [4].

Class I PHA polymerases use short chain length (scl) thioesters (consisting of 3-5 carbons) as substrate. The best studied enzyme in this class is the PHA polymerase of *Ralstonia eutropha *[5,6]. This enzyme has been suggested to be active as a homodimer [7,8]. Class III and Class IV polymerases are represented by the PHA polymerase of *Allochromatium vinosum *and *Bacillus megaterium*, respectively, both of which consist of two different subunits [9,10]. Similar to class I enzymes, 3-hydroxybutyryl-CoA is the preferred substrates. It has, however, been shown by *in vivo *and *in vitro *studies that Class I and Class III polymerases also have a slight affinity for medium chain length (mcl) 3-hydroxyacyl-thioesters [11-13]. Class II polymerases represent enzymes with a high affinity for medium chain length (mcl) 3-hydroxyacyl-thioesters (C6-C14) and are mainly found in *Pseudomonas *species [14,15]. Two exceptions to this classification are the PHA polymerases of *Thiocapsa pfennigii *and *Aeromonas caviae*. These enzymes have a substrate range that includes both scl- and shorter mcl-CoA thioesters [16,17].

*Pseudomonas putida *GPo1 and many other *Pseudomonas *species contain two different class II polymerases, encoded by *phaC1 *and *phaC2 *[18,19]. Alignment of the polymerase sequences shows clear differences [3], so that functional differences between these polymerases could be envisaged. In *P. putida*, both polymerases are active independently of one another, and *in vivo *studies have shown only small differences between these two enzymes [20-23]. It remains unclear what metabolic advantages *P. putida *derives from having two PHA polymerases. Although numerous studies have been carried out on PHA polymerases and they are well characterized at the molecular level [24,25], the biochemical properties of the class II polymerases have not been studied in detail, mainly due to the difficulty in obtaining the purified proteins. Previously the polymerases have been purified to homogeneity, however, the activities of the purified enzymes were several magnitude lower than the granule-bound enzymes [26-28]. It is problematic to study the intrinsic properties of these enzymes with such low activities, although they are pure.

Thus, in this report we investigated the activities of the granule-bound polymerases. Both enzymes were (partially) purified and a wide range of 3-hydroxyacyl-CoA precursors were prepared, allowing the study of substrate range and kinetic parameters. In addition, the release of both PHA polymerases from the granule was investigated using various detergents. Based on the characteristics of granule release and the activity profiles of these two enzymes we conclude that there are substantial functional differences between granule bound PhaC1 and PhaC2.

## Results

### Overexpression of PHA polymerases

**Figure 1:**
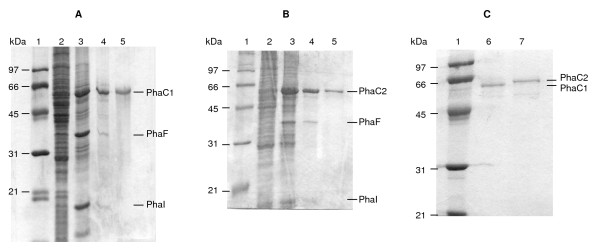
**SDS-PAGE of different fractions following purification of PhaC1 (A) and PhaC2 (B) and comparison of native PhaC1 and PhaC2 (C)**. Lanes: 1, molecular mass standards (kDa); 2, crude extract of *P. putida *GPp104 carrying pGEc405 (A) or pGEc404 (B); 3, isolated PHA granules; 4, released proteins using 0.5% (v/v) Triton X-100 (A) or 0.1% (w/v) rhamnolipids (B); 5, purified PhaC1 (A) and PhaC2 (B) after Source15Q chromatography; 6, purified native PhaC1 (C); 7, purified native PhaC2 (C).

Previously it has been found that the PHA polymerases attached to PHA granules from wild-type *P. putida *GPo1 were only present at low amount and could not be detected as a distinct protein band in SDS-PAGE analysis relative to total protein [29]. When PHA granules of GPo1 were purified and analyzed by SDS-PAGE, only PhaC1 was visible, whereas PhaC2 was not detectable [30]. To study activities of both polymerases, *phaC1 *and *phaC2 *were overexpressed from pGEc405 (*phaC1*) and pGEc404 (*phaC2*) [20], and were transformed, respectively, into *P. putida *GPp104, a non-PHA producer [20]. With octanoate as substrate these transformants produced 32% and 10% PHA relative to total cell dry mass, respectively, demonstrating that both *phaC1 *and *phaC2 *expressed active proteins. Isolated PHA granules from *P. putida *GPp104 [pGEc405] contained a major protein band of about 60 kD (Fig. 1A, lane 3). Similarly, a protein band of about 64 kD was observed in *P. putida *GPp104 [pGEc404] (Fig. 1B, lane 3). N-terminal sequencing revealed that the 60 kD protein is PhaC1 and 64 kD protein is PhaC2. No PhaC2 was detected from isolated GPp104 [pGEc405] granules (Fig. 1A), and no PhaC1 was found from GPp104 [pGEc404] granules (Fig. 1B). Thus, GPp104 in combination with pGEc404 or pGEc405 is a suitable candidate for studying PhaC1 and PhaC2 individually.

### Purification of PhaC1 and PhaC2 from PHA granules

PHA granules contain a number of bound proteins including PhaC1, PhaC2, the PHA depolymerase, acyl-CoA synthetase and the previously identified structural proteins PhaF and PhaI (Fig. 1, [30,31]). Several detergents were tested for their ability to specifically release the PHA polymerases. As found earlier [32], PhaC1 was released efficiently with most of the tested detergents, in contrast to other granule-associated proteins that remained predominantly granule-bound (Table 1). Triton X-100 (0.5% w/v) was chosen as the most suitable detergent to preferentially release PhaC1 from PHA granules (Fig. 1A, lane 4). PhaC2 bound much more strongly to the PHA granule than PhaC1; among the detergents tested only rhamnolipids (0.1% w/v) released the enzyme from PHA granules (Fig. 1B, lane 4). However, rhamnolipids also released other granule-associated proteins (Table 1).

**Table 1:** Effect of different detergents on the release of PhaC1, PhaC2 and other proteins from PHA granules.

Extractant (w/v)^a^	PhaC1	PhaC2	PhaF	PhaI
	Proteins released from PHA granules (%)^b^			
Tris-HCl pH 8 (100 mM)	n.d^c^	n.d	n.d	n.d
Phospholipids (0.5%)	n.d	n.d	n.d	n.d
Hecameg (0.5%)	50%	n.d	n.d	10%
Triton X-100 (0.5%)	100%	0-20%	10%	10%
CHAPS (0.5%)	100%	0-20%	n.d	25%
Igepal-CA630 (0.5%)	100%	0-20%	n.d	25%
Rhamnolipids (0.1%)	100%	100%	75%	75%

a) Detergents were dissolved in 100 mM Tris-HCl (pH 8).

b) Percentages of protein release were determined by comparing granule pellet fractions and supernatant fractions on SDS-PAA gels.

c) Not detected (< 2% of total granule bound protein).

PhaC1 and PhaC2 were purified further (~90%) by anion-exchange chromatography over a Source 15Q column, eluted with 50 mM KPi, pH 7 using a 0 - 0.5 M NaCl gradient. This also resulted in the removal of detergent which is inhibitory to the PHA polymerase activities [17,33]. Both proteins were found to bind strongly to the anion exchange column, suggesting that these proteins have net negative surface charges at pH 7. This was surprising given their sequence based isoelectric points (6.9 and 9.1 for PhaC1 and PhaC2, respectively). A more likely explanation is that the hydrophobic PhaC1 and PhaC2 bound to the polystyrene/divinyl benzene matrix of the 15Q beads, and that this hydrophobic interaction was dominant over the ionic interactions.

The purified polymerase fractions could not be concentrated by using protein filters, as both PhaC1 and PhaC2 adsorbed to the polyethersulfone membranes (Millipore). The purified fractions were therefore precipitated with ammonium sulfate and subsequently dissolved in a small volume of Tris buffer and dialyzed. The purification of PhaC1 and PhaC2 was followed by SDS-PAGE (Fig. 1A and 1B). Purified PhaC2 was slightly larger than PhaC1 (Fig. 1C), in agreement with earlier results [32] and the calculated molecular weights of 62.4 kD and 62.6 kD for PhaC1 and PhaC2, respectively.

### Activities of PhaC1 and PhaC2

The activities of PHA polymerases were measured using the consumption of 3-hydroxyoctanoyl-CoA or the release of free CoA. For activity measurements (acyl-CoA consumption) in crude cell extracts, a modified assay was used in which free CoA (1 mM) was added to the assay mixture. This completely eliminated the action of thioesterases which were found to interfere with the activity assay by hydrolyzing the substrate (3-hydroxyoctanoyl-CoA) and had no significant influence on PhaC activities [30]. Table 2 shows the measured activities in the various purification steps for PhaC1 and PhaC2. Despite adequate recovery of both PhaC1 and PhaC2 (61% and 50% respectively) only minor activity (two to three orders of magnitude lower than that of the granule bound proteins) could be recovered when the enzymes were purified to homogeneity. The loss of activity was observed upon solubilization of PhaC1 and PhaC2 from the granule by detergents. Activity could not be re-established by removal of the detergent using a SourceQ anion exchange column. Size exclusion chromatography suggested an approximate molecular mass of 65 kD for both purified PhaC1 and PhaC2 indicating that the enzymes were eluted in the monomeric form (result not shown). As the active enzyme may occur in a dimeric or multimeric form, we attempted to reconstitute the enzyme activity by addition of sugars (50% glycerol, glucose, mannose or fructose), presumed primers (0.25 mM 3-hydroxydecanoyl-3-hydroxydecanoyl-CoA), phospholipids (1 mg/ml) or PHA granules (1 mg/ml amorphous or boiled). None of these approaches were successful (data not shown).

**Table 2:** Purification of PhaC1 and PhaC2 from *P. putida *GPp104 [pGEc405] and GPp104 [pGEc404].

Fraction		Total protein (mg)	PhaC (mg)	Recovery of PhaC (%)	Total activity (U)	Specific activity (U/mg protein)	Specific activity (U/mg PhaC)	Recovery of total activity (%)
Crude cell extract	PhaC1	750	1.8^a^	-^c^	67.5^b^	0.09^b^	37.5	100
	PhaC2	850	-^c^	-^c^	3.2^b^	0.0375^b^	-^c^	100
Isolated PHA granules	PhaC1	2.4	1.3	100	52.8	22	40.6	78
	PhaC2	2.6	1.4	100	2.9	1.1	2.1	91
Released proteins from PHA granules	PhaC1	1.5	1.1	85	<0.01	<0.01	<0.01	<0.01
	PhaC2	1.2	0.9	64	<0.01	<0.01	<0.01	<0.01
Source 15Q and ammonia precipitation	PhaC1	0.9	0.8	61	< 0.01	< 0.01	<0.01	<0.01
	PhaC2	0.7	0.7	50	<0.01	<0.01	<0.01	<0.01

a) The amount of PhaC1 was estimated by Western blotting using anti-PhaC1 antibodies. In all other fractions, PhaC1 was estimated by densitometric scanning.

b) Activity was assayed by measuring depletion of 3-hydroxyoctanoyl-CoA (HPLC) in the presence of 1 mM CoA (to reduce background activity). In all other fractions PhaC1 activity was measured spectrophotometrically (A412) by following the release of CoA using DTNB.

c) Not determined.

From the data presented in Table 2 as well as in Fig. 1 it seems that either PhaF or PhaI (or both) might contribute to PhaC activity: the more of these phasins are removed from PhaC1 or PhaC2 the more enzyme activity is lost. In order to investigate whether a specific phasin is responsible for polymerase activity, we tested *P. putida *GPo1 mutants which lack of either PhaF (*P. putida *GPG-Tc-6) [34] or PhaI (*P. putida *GPo1001) [35]. The PhaF-negative granules did not show a reduction of PhaC activity compared to granules from the parental strain. An about 1.5 fold reduction of PhaC activity could be determined for PhaI-negative granules. These results indicate that PhaI has more impact on PhaC activity than PhaF. Nevertheless these data demonstrate also that phasins are not essential for PHA polymerase activity or PHA synthesis, similar to what has been reported previously [36].

### Determination of enzyme kinetics of PHA polymerases attached to the PHA granules

In order to determine enzyme kinetics, activities of both granule-bound PhaC1 and PhaC2 were monitored spectrophotometrically by following the release of CoA. The substrate *R*-3-hydroxyoctanoyl-CoA concentrations ranged from 0.0375 to 0.25 mM. The correlation of reaction velocity with the substrate concentration could be fitted to Michaelis-Menten kinetics with the aid of nonlinear regression analysis (Sigma Plot enzyme kinetics; systat software, Inc.). A *Km *of 125 (± 35) μM and a *V*max of 40.8 (± 6.2) U/mg PhaC1 could be derived for the granule-bound PhaC1, a *Km *of 37 (± 10) μM and a *V*max of 2.7 (± 0.7) U/mg PhaC2 could be derived for the granule-bound PhaC2 (Table 3). Surprisingly, the *V*max of PhaC2 was more than 15-fold lower than that of PhaC1. This suggests that PhaC1 and PhaC2 might have different substrate spectra, which were examined as described below.

**Table 3:** Kinetic parameters of granule-bound PhaC1 and PhaC2 towards *R*-3-hydroxyoctanoyl-CoA.

	Km (μM)	Vmax (U/mg PhaC)
Granule-bound PhaC1	125 ± 35	40.8 ± 6.2
Granule-bound PhaC2	37 ± 10	2.7 ± 0.7

- Kinetic parameters were determined from initial PHA polymerase activities which were determined spectrophotometrically (A412) by following the release of CoA using DTNB.

- PHA granules were isolated from *P. putida *GPo1 after 16 hours of growth.

- Values are the average of 4 determinations.

### Substrate specificity of granule-bound PhaC1 and PhaC2

**Figure 2:**
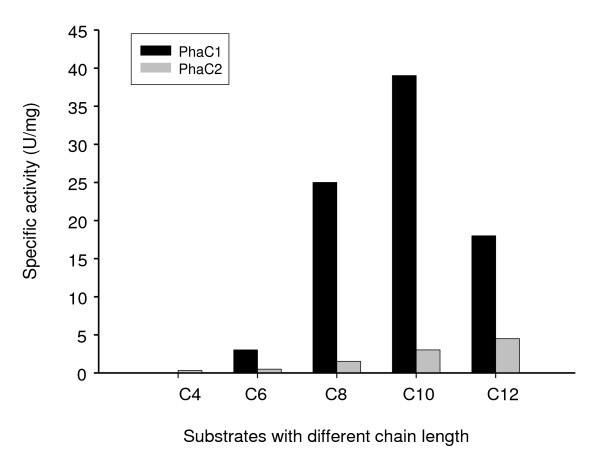
**Substrate specificity of granule-bound PhaC1 and PhaC2**. Assay conditions: 100 mM Tris-HCl pH 8, 1 mg/ml BSA, 0.5 mM MgCl_2_, 0.2 μg/ml granule bound PhaC1 or 2 μg/ml granule bound PhaC2 with 0.25 mM of one of the following acyl-CoA substrates: *R/S-*3-hydroxybutyryl-CoA (C4), *R-*3-hydroxyhexanoyl-CoA (C6), *R-*3-hydroxyoctanoyl-CoA (C8), *R-*3-hydroxydecanoyl-CoA (C10) and *R-*3-hydroxydodecanoyl-CoA (C12). Initial activity was measured spectrophotometrically (A412) by following release of CoA using DTNB.

Various 3-hydroxyacyl-CoA thioesters differing in side chain length were synthesized and tested on both granule-bound PhaC1 and PhaC2. Fig. 2 shows that PhaC1 has a preference for medium chain length 3-hydroxyacyl-CoA esters with highest affinity towards 3-hydroxydecanoyl-CoA (41 U/mg PhaC1). Very little activity is observed towards 3-hydroxybutyryl-CoA (< 1% of the activity seen with 3-hydroxydecanoyl-CoA). Moderate activity was found for 3-hydroxyhexanoyl-CoA and high activity was found for 3-hydroxyoctanoyl-, 3-hydroxydecanoyl- and 3-hydroxydodecanoyl-CoA. PhaC2 on the other hand seemed to have a much broader specificity towards substrates varying from 3-hydroxybutyryl- up to at least 3-hydroxydodecanoyl-CoA. Activity increased with longer side chains of the tested substrates with a maximum activity of 4.5 U/mg PhaC2 for 3-hydroxydodecanoyl-CoA. The activities towards substrates with longer chain length (above C12) could not be determined due to the unavailability of the substrates. In general, PhaC1 exhibited 5-10 fold higher activities than PhaC2 towards the tested substrates (Fig. 2).

## Discussion

PHA polymerases have been identified for several decades. We and other groups have previously purified the polymerases from different *Pseudomonas *strains [26-28]. However, investigation of class II polymerases has thus far been hampered by low activities of the purified enzymes and a lack of readily available 3-hydroxyacyl-CoA precursors [26-28]. Here we report the first characterization of PHA granule-bound PhaC1 and PhaC2 from *P. putida *GPo1. Granule-bound PhaC1 and PhaC2 were obtained by overexpression of either *phaC1 *or *phaC2 *in the PHA deficient host *P. putida *GPp104 and subsequent isolation of PHA granules after growth on octanoate. A series of 3-hydroxyacyl-CoA substrates was synthesized allowing determination of substrate specificity and kinetic parameters of both granule bound PhaC1 and PhaC2.

Both PhaC1 and PhaC2 were purified using anion-exchange chromatography. However, both enzymes lost their activities during purification to electrophoretic homogeneity after release from the PHA granules (Table 2). It has been reported that purified PHA polymerases exist in an equilibrium of monomeric and dimeric forms, and dimerization is significantly induced in the presence of substrate [26-28]. Thus, various attempts were undertaken to reconstitute the activities, such as addition of intact PHA granules, postulated primer (3-hydroxydecanoyl-3-hydroxydecanoyl-CoA) and phospholipids, however, none of these additives had a positive effect. Based on their amino acid sequence both PhaC1 and PhaC2 are not exceptionally hydrophobic. Upon release from PHA granules, however, the PhaCs were found strongly sticking to various surfaces (e. g. polystyrene, polyethersulfone). Thus, it can not be excluded that the PHA polymerases are (partially) denatured after release from the granules after which the hydrophobic core becomes surface exposed. Another reason for the activity loss of the purified enzymes could be that some low molecular weight modulating compound(s) gets lost during the purification processes.

Previously it has been proposed that PHA polymerase 1 of *P. aeruginosa *PAO1 was covalently attached to the PHA granules because the polymerase could not be released from PHA granules by 8 M urea [37]. The polymerases of PAO1 and GPo1 share 80% identity at the amino acid level. It was therefore unexpected that PhaC1 of GPo1 could be readily released from PHA granules by mild detergent treatment (Table 1, Fig. 1A), and thus rather proposing that PhaC1 interacts with PHA granules through other forces than covalent binding. It is not clear what causes this difference.

Since only a few PHA polymerases have been purified to homogeneity, the substrate specificity of almost any polymerase can only be estimated from cultivation experiments with precursor substrates provided as carbon source. The subsequent analysis of the monomer composition of the accumulated PHA was used as a measure of the *in vivo *substrate specificity [4]. Such analysis has limitations because some bacteria, such as pseudomonads, contain more than one PHA polymerase gene, and the physiological background in which the available precursors for PHA polymerase may vary considerably. In this study, expressing *phaC1 *or *phaC2 *in a PHA-negative mutant allowed the independent investigation of the class II PHA polymerases PhaC1 and PhaC2 from *P. putida*. It was found that the characteristics of granule-bound PhaC1 and PhaC2 differ more than was previously assumed [20,21]. First, PhaC2 has a much higher affinity for PHA granules than PhaC1. Whereas mild non-ionic detergents such as Triton X-100, CHAPS and Igepal CA-630 efficiently released PhaC1 from the native granules, PhaC2 was only released by rhamnolipids. This natural detergent was probably more effective than the mild detergents in releasing PhaC2 from the native granules due to its structural similarity with mcl-PHA [38]. Second, there was a significant difference in the substrate specificity of granule-bound PhaC1 and PhaC2. PhaC1 exhibited high activity towards medium chain length 3-hydroxyacyl thioesters, whereas PhaC2 demonstrated a much broader substrate range towards substrates with chain lengths varying from C4 to at least C12 residues. Earlier *in vivo *experiments showed only slight differences in polymer composition for PHA synthesized with the two polymerases [20,21,39]. More recent work with *Pseudomonas stutzeri *[40,41] and *Pseudomonas mendocina *[39] has shown that *in vivo *PhaC2 is more active with short chain compared with long chain length 3-hydroxyfatty acids. Given the higher activity of the granule bound PhaC2 with long rather than with short chain 3-hydroxyfatty acids shown here, these previous observations suggest that the PHA composition found *in vivo *is determined predominantly by the available intracellular 3-hydroxyacyl-CoA intermediates rather than by the inherent specificity of the PHA polymerases.

The observed difference in substrate specificity of PHA granule bound PhaC1 and PhaC2 in this work is likely caused by the intrinsic properties of the two PhaCs, especially when considering that PhaC1 and PhaC2 share 53% identity at the amino acid level. However, we cannot rule out the possibility that other PHA granule bound proteins/compounds have influence on the PhaC activity and impact the activities of PhaC1 and PhaC2 to a different extent.

## Conclusion

Although molecular analysis of the class II PHA polymerases has provided information on catalytic mechanism (see review [4]), much research still has to be undertaken at the biochemical level of these enzymes. Here we studied and compared the activities and substrate specificities of PHA granules bound PhaC1 and PhaC2 by overexpressing *phaC1 *and *phaC2*, respectively, in a PHA-negative mutant. It was found that release of polymerases from PHA granules led to activity loss and the activities could not be recovered despite extensive efforts. For future studies it will be necessary to obtain soluble enzyme constructs with comparable activities to the PHA granule-bound polymerases. This will allow structure analysis as well as facilitate the production of tailor-made PHA and *in vitro *synthesis of PHA.

## Methods

### Materials

*R-*3-hydroxyalkanoic acids were prepared by hydrolysis of mcl-PHA [42], *R/S-*3-hydroxyalkanoic acids were supplied by Larodan Lipids (Malmö, Sweden), and *R/S-*hydroxybutyryl-CoA was supplied by Sigma (St. Louis, US). Several other 3-hydroxyacyl-CoA derivatives were synthesized by enzymatic coupling of CoA to *R- *or *R/S-*3-hydroxyalkanoic acids catalyzed by acyl-CoA synthetase. Reaction conditions were as described previously [29,43].

*R- *or *R/S-*3-hydroxyacyl-CoA was purified from the reaction mixture using a preparative C18-RP column together with a preparative chromatography system (Waters Prep LC™ 486). A linear gradient starting from 10% (v/v) methanol and 90% NH_4_Ac pH 5.5 (50 mM) to 100% methanol was used to elute the 3-hydroxyacyl-CoA esters. *R-*3-hydroxyvaleryl-CoA, *R-*3-hydroxyhexanoyl-CoA, *R-*3-hydroxyoctanoyl-CoA, *R-*3-hydroxydecanoyl-CoA and *R-*3-hydroxydodecanoyl-CoA, eluted at retention times of 10 min (24% methanol), 13 min (40%), 20 min (70%), 24 min (92%) and 27 min (100%), respectively. The molecular weight of each thioester was confirmed by LC-MS. Volatile methanol was immediately evaporated by vacuum rotation (Rotavap, Büchi, Switzerland). The remaining NH_4_Ac was evaporated by freeze drying. Purified freeze-dried 3-hydroxyacyl-CoAs were stable for at least 6 months at -20°C.

*R-*3-hydroxydecanoyl-3-hydroxydecanoate was prepared by hydrolytic cleavage of the glycosidic bonds in rhamnolipids [38]. Rhamnolipids (1 g) were prepared as described previously [38] and were incubated with 50 ml dioxane and 50 ml HCl (1 M) for 1 h at 100°C. The released 3-hydroxydecanoyl-3-hydroxydecanoate group was extracted with ethyl-ether and subsequently dried using vacuum evaporation (Rotavap, Büchi, Switzerland). The purified 3-hydroxydecanoyl-3-hydroxydecanoate was dissolved in 200 mM KPi pH 7 and activated with CoA using acyl-CoA synthetase (Sigma) according to a protocol described previously [43].

The concentration of all prepared CoA esters was estimated by hydroxylamine treatment [44], which causes the release of bound CoA. The concentration of free CoA before and after hydroxylamine treatment was analyzed by the Ellman method [45].

### Bacterial growth and PHA production

*P. putida *GPp104 (PHA-negative) was transformed with either pGEc405 or pGEc404 which encode for *phaC1 *and *phaC2*, respectively [20]. *P. putida *GPo1 mutants which lack either PhaF (*P. putida *GPG-Tc-6) [34] or PhaI (*P. putida *GPo1001) [35] were also used. The strains were precultured in Luria-Bertani medium prior to large-scale cultivation. In order to stimulate PHA accumulation, the recombinants were cultivated in 0.2 NE_2 _medium (mineral medium containing 20% of the total nitrogen of E2 medium) supplemented with 15 mM sodium octanoate, 0.1% yeast extract and 50 μg/ml of kanamycin. Cells were grown in a fermenter (24 l MBR reactor) as previously described [33]. After 20 h, cells were harvested by centrifugation at 4,500 g for 15 min at 4°C and stored in small batches at -80°C. PHA content and composition of freeze-dried cells were measured after methanolysis according to a GC method described previously [46].

### Purification of PhaC1 and PhaC2

Osmotically sensitive cells of *P. putida *GPp104 transformants (1.5 g dry biomass) were prepared as described before [47]. Spheroplasts were resuspended in H_2_O to a final concentration of 50 mg/ml and disrupted by two passages through a pre-cooled French pressure cell.

Isolation of PHA granules: PHA granules were isolated by density centrifugation [33] according to a procedure which was modified for larger volumes of cell lysate. Broken cells (50 mg/ml) (30 ml) were loaded on top of a 20% sucrose layer (200 ml) and subsequently centrifuged (15,000 g) for 3 hours. The PHA granules, which remained on top of the sucrose layer, were collected and washed twice with 100 mM Tris-HCl pH 8. The final PHA pellet was resuspended in 30 ml of 100 mM Tris-HCl pH 8.

Release of PhaC1 and PhaC2 from PHA granules: PhaC1 and PhaC2 were released from the granules by incubation with detergents in 100 mM Tris-HCl pH 8 (4°C, 30 min) using a table shaker (1,000 rpm). The following detergents were tested: phospholipids (0.5% w/v), Hecameg (0.5% w/v), Triton X-100 (0.5% w/v), CHAPS (0.5% w/v), Igepal-CA630 (0.5% w/v) and rhamnolipids (0.1% w/v). Out of the tested detergent Triton X-100 (0.5% w/v) was used for removal of PhaC1, while rhamnolipids (0.1% w/v) were used for the release of PhaC2.

Anion-exchange chromatography: After pelleting the PHA granules by centrifugation (12,000 g, 30 min), the supernatant (containing the released proteins) was applied on a Source 15Q column (PE 4.6/100, Pharmacia) which was equilibrated with 50 mM KPi pH 7 + 5% (v/v) glycerol. After loading, the column was washed with 5 volumes of 50 mM KPi pH 7 + 5% (v/v) glycerol in order to remove the detergents. Proteins were eluted using a 0-500 mM NaCl gradient (in 50 mM KPi pH 7 + 5% (v/v) glycerol). Fractions (2 ml) were collected. PhaC1 eluted between 350 and 400 mM NaCl whereas PhaC2 eluted between 300 and 350 mM NaCl. The pooled fractions containing either PhaC1 or PhaC2 were concentrated by ammonium-sulfate precipitation. The fractions were diluted with 1 volume of a saturated (NH_4_)_2_SO_4 _solution and incubated for 30 minutes on ice. After centrifugation (24,000 g, 30 min), the pellet was resuspended in 10 mM Tris-HCl pH 8 and dialyzed overnight against 10 mM Tris-HCl pH 8. The final extract was stored in 50% (v/v) glycerol at -20°C.

### PHA polymerase activity assay

PHA polymerase activity was analyzed by following the release of CoA using DTNB. A typical mixture (300 μl) contained 0.5 mM *R*-3-hydroxyoctanoyl-CoA, 0.2 μg/ml of granule-bound PHA polymerase (or 25 μg/ml of soluble PHA polymerase), 1 mg/ml BSA and 0.5 mM MgCl_2 _in 100 mM Tris-HCl pH 8. Activity was measured spectrophotometrically as previously described [33]. For enzyme characterization 3-hydroxyoctanoyl-CoA was used as substrate at a range of 0.0375 and 0.25 mM. One unit is defined as 1 μmol *R*-3-hydroxyoctanoyl-CoA consumption per minute. Values presented here are the average of 4 determinations.

PHA polymerase activity in crude cell extracts was measured by following the depletion of *R-*3-hydroxyoctanoyl-CoA using HPLC [43]. A typical reaction mixture contained 0.5 mM *R*-3-hydroxyoctanoyl-CoA, 1 mM CoA, 0.1 mg/ml crude cell extract, 1 mg/ml BSA and 0.5 mM MgCl_2 _in 100 mM Tris-HCl pH 8.

### Protein determination

The amount of PhaC1 and PhaC2 in the various purification fractions was estimated by densitometric scanning of SDS-PAA gels using a Multimage™Light Cabinet (Alpha Innovation Corp.) with chemiluminescence and visible light imaging. Various purification fractions were compared to known amounts of BSA. In crude cell extracts, PhaC1 concentrations were determined by Western blotting as described previously [29].

### LC-MS analysis

The molecular mass of the synthesized *R*-3-hydroxyacyl-CoAs was confirmed by liquid chromatography (LC) mass spectrometry (MS) analysis using negative spray ionization (Agilent 1100 series). The MS settings were as follows: atmospheric pressure chemical ionization mode; negative ionization, fragmentor voltage, 50 V; gas temperature, 350°C; vaporizer temperature, 375°C; drying gas (N2), flow rate, 4 liter min-1; nebulizer pressure, 0.023 N/m^2^; capillary voltage, 2,000 V; corona current, 6 μA.

## Abbreviations

For ease of readability, the following abbreviations were used: PHA: polyhydroxyalkanoate; Mcl: medium chain length; Lcl: long chain length; PhaC1: PHA polymerase 1; PhaC2: PHA polymerase 2; BSA: bovine serum albumin; CoA: coenzyme A; DTNB: 5,5'-dithiobis(2-nitrobenzoic acid); LC-MS: liquid chromatography - mass spectrometry; HPLC: high performance liquid chromatography; SDS-PAGE: sodium dodecyl sulfate poly acrylamide gel electrophoresis; Hecameg: methyl-6-O-(N-heptylcarbamoyl)-α-D-glucopyranoside; CHAPS: 3-[(3-cholamidopropyl)dimethylammonio]-1-propanesulfonate; Igepal-CA630: polyethylene glycol octylphenol ether.

## Competing interests

The authors declare that they have no competing interests.

## Authors' contributions

QR and GdR performed the laboratory experiments and drafted the manuscript. BW adviced the experimental design and revised the drafted manuscript. MZ and LTM helped in revision of the manuscript. All authors read and approved the final manuscript.
